# Editorial: Current status of fruit tree improvement through biotechnology

**DOI:** 10.3389/fpls.2024.1472444

**Published:** 2024-08-07

**Authors:** Cristian Silvestri, José Ángel Mercado, Elena Palomo-Ríos

**Affiliations:** ^1^ Dipartimento di Scienze Agrarie e Forestali (DAFNE), Università della Tuscia, Viterbo, Italy; ^2^ Departamento de Botánica y Fisiología Vegetal, Instituto de Hortofruticultura Subtropical y Mediterránea “La Mayora”, Universidad de Málaga, Spanish National Research Council (IHSM-UMA-CSIC), Málaga, Spain

**Keywords:** fruit trees, genetic improvement, fruit tree biotechnology, transgenic research, genome editing

Climate change exerts a significant impact on crop production, increasing the number of biotic and abiotic stresses plants must face. This factor, jointly with the increase in world population and the reduction of land available for agriculture, poses a challenge to the sustainable production of fruit tree crops. Novel agricultural technologies are required to minimize these threats and fulfill the increasing demand for fruits. Fruit tree breeding programs focus on issues such as higher tolerance against abiotic stress, disease resistance, fruit quality, and postharvest shelf life; however, breeding programs are hampered by the long juvenile phase of trees, which hinders the process and lengthens the release of improved genotypes. Novel biotechnology approaches offer huge opportunities to enhance the resilience, productivity, and quality of fruit trees. Our Research Topic, “Current Status of Fruit Tree Improvement Through Biotechnology,” brings together studies that highlight the innovative use of biotechnological tools and approaches in fruit tree research and innovation. This editorial synthesizes the findings from these contributions, highlighting key themes and implications for the future of fruit tree breeding.

## Advancing disease resistance in fruit trees

A significant focus of the research collected here is on combating diseases that threaten fruit tree health and productivity. Jia et al. demonstrated a breakthrough in citrus canker resistance by employing the Cas12a/CBE co-editing method to precisely target the susceptibility gene LOB1 in *Citrus sinensis* cv. Hamlin. This approach not only resulted in canker-resistant plants but also achieved remarkable efficiency in generating transgene-free lines, which is crucial for regulatory concerns and consumer acceptance. Similarly, Spencer et al. explored the use of CRISPR/Cas13 technology to confer resistance to grapevine virus A (GVA) in *Vitis vinifera*. By targeting the viral RNA, they developed a promising strategy to solve viral diseases in grapevines, which pose significant threats to grape production worldwide. These studies emphasize how biotechnological innovations can provide sustainable solutions to disease management in fruit crops while also promoting low environmental impact. Alburquerque et al. explored trans-grafting as a strategy to transfer virus resistance from transgenic plum rootstocks to apricot scions. Their findings highlighted the effectiveness of RNA-silencing-based resistance in protecting stone fruits from viral diseases, offering a practical solution while addressing public concerns about genetically modified organisms (GMOs). This approach represents a paradigm shift in disease management strategies, emphasizing the potential of grafting technologies in fruit tree orchards.

## Enhancing breeding efficiency and developmental traits

Another critical area of exploration is the enhancement of breeding efficiency and manipulation of developmental traits in fruit trees. Guerrero et al. addressed the challenge of the long juvenile phase in olive trees by characterizing olive plants transformed with a flowering locus T (FT) gene from *Medicago truncatula*. The expression of this gene accelerated flowering in olives, offering a pathway to shorten breeding cycles and the obtainment of new cultivars adapted to changing climatic conditions and to meet market demands. Tomes et al. applied a similar approach in European pear (*Pyrus communis*) using a MADS-box gene to induce early flowering. Their findings not only advanced the understanding of floral development in pears but also highlighted the potential to accelerate breeding programs across various fruit tree species. Both studies highlight how biotechnology can revolutionize traditional breeding, facilitating the rapid development of novel varieties with improved traits.

## Addressing challenges in orchard production and sustainability

Kerr et al. provided a comprehensive review of the challenges facing orchard production, particularly in the context of climate change and sustainability. They emphasized the importance of identifying resilient traits and adopting innovative technologies to future-proof orchard systems against environmental stresses and emerging pests. By integrating genomic tools and molecular techniques, researchers can uncover genetic markers for resilience and productivity, paving the way for sustainable orchard management practices. Gill et al. discussed the potential of using transgenic rootstocks to confer tolerance to Huanglongbing (HLB) in citrus. Their review highlighted the role of rootstock-mediated mechanisms in enhancing tree health and resilience to diseases, offering an alternative approach to traditional breeding methods. These insights not only address critical disease challenges but also contribute to the development of eco-friendly and sustainable solutions in fruit tree cultivation.

## Harnessing genomic tools for precision breeding

Advances in genomic tools and molecular techniques are central to the progress in fruit tree improvement addressed in this Research Topic. Deng et al. conducted a comprehensive genome-wide analysis of the GH9 gene family in mulberry, elucidating key genes involved in fruit abscission. Their findings provide insights into genetic mechanisms controlling fruit fall, which can inform strategies to enhance fruit retention and yield in mulberry cultivation. Pavese et al. pioneered the use of CRISPR/Cas9 ribonucleoprotein (RNP) technology in holm oak (*Quercus ilex*), demonstrating precise genome editing without the integration of foreign DNA. This approach holds promise for developing disease-resistant varieties and addressing ecological challenges in Mediterranean ecosystems where holm oak plays a vital role. These studies underscore the potential of genomic tools in advancing fruit tree breeding towards sustainability and resilience.

## Conclusion

The manuscripts collected in this Research Topic collectively illustrate the transformative impact of biotechnology on fruit tree improvement. From precise gene editing and disease resistance strategies to innovative breeding techniques and sustainable orchard management practices, these studies exemplify how biotechnological innovations are reshaping the future of fruit cultivation. As these technologies continue to evolve and integrate with traditional breeding methods, they hold immense promise for addressing global food security challenges and ensuring the sustainability of fruit tree production in a changing world.

## Author contributions

CS: Writing – original draft, Writing – review & editing. JM: Writing – original draft, Writing – review & editing. EP-R: Writing – original draft, Writing – review & editing.

